# Spinal dysraphism associated with OEIS complex: aspects of diagnosis and treatment

**DOI:** 10.1186/1743-8454-7-S1-S6

**Published:** 2010-12-15

**Authors:** Cora Städtler, Reinhold Cremer, Maximilian W Kellner, Thomas M Boemers

**Affiliations:** 1 Children’s Hospital of Cologne, Municipal Hospital of the City of Cologne, Amsterdamer Str. 59, 50735 Cologne, Germany

## Background

Associated neurospinal dysraphism in combination with anomalies of the genitourinary tract is well known in children with imperforate anus. There is a broad spectrum of concomittant anomalies with the OEIS Syndrome as the most serious endpoint. OEIS is an acronym for omphalocele, exstrophy (bladder exstrophy/cloacal exstrophy), imperforate anus and spinal defects. At present almost all cases of OEIS are diagnosed prenatally and the patients should be treated at a specialized center with a multidisciplinary team. The complex diagnostic procedures and surgical measures are addressed as well as the difficulties in achieving continence in these patients.

## Materials and methods

We present data of six patients with imperforate anus and spinal dysraphism (4 with meningomyelocele, two with lipomeningocele) currently managed in the spina bifida clinic of the Children’s Hospital of Cologne. Two of them presenting the complete OEIS syndrome symptoms, another two with cloacal exstrophy without omphalocele and two with major renal malformations. Only two of six patients had hydrocephalus.

## Results

The often difficult and multiple reconstructive surgical procedures in these patients are described (e.g. creation of neo-bladder with catheterizable channel, colostomy, pull-through procedures, closure of omphalocele and neurosurgical procedures). Especially the management of incontinence is extremely complex in these patients. For instance there is no sufficient bladder capacity in patients following initial closure of bladder exstrophy and there is only a weak or no function of the anal sphincter following pull-through procedures.

## Conclusions

OEIS syndrome is a devastating combination of anomalies resulting in a neurological deficit similar to myelomeningocele. In contrast to the functional problems arising in spina bifida patients, children with OEIS syndrome have functional impairment with additional problems due to the malformed urogenital tract and anorectal system. Management of children with OEIS syndrome demands a careful management plan starting already in the prenatal period and resulting in a life-long surveillance. Treatment of these patients should only be performed by a multidisciplinary team in a specialized center.

